# Accumulation of Oxidized Low-Density Lipoprotein in Psoriatic Skin and Changes of Plasma Lipid Levels in Psoriatic Patients

**DOI:** 10.1155/2007/78454

**Published:** 2006-12-27

**Authors:** Nilgun Solak Tekin, Ishak Ozel Tekin, Figen Barut, Emine Yilmaz Sipahi

**Affiliations:** ^1^Department of Dermatology, Faculty of Medicine, Zonguldak Karaelmas University, Kozlu, 67600 Zonguldak, Turkey; ^2^Department of Immunology, Faculty of Medicine, Zonguldak Karaelmas University, Kozlu, 67600 Zonguldak, Turkey; ^3^Department of Pathology, Faculty of Medicine, Zonguldak Karaelmas University, Kozlu, 67600 Zonguldak, Turkey; ^4^Department of Pharmacology, Faculty of Medicine, Zonguldak Karaelmas University, Kozlu, 67600 Zonguldak, Turkey

## Abstract

*Background*. Psoriasis is a chronic inflammatory skin disease characterized by an accelerated turnover of epidermal cells and an incomplete differentiation in epidermis with lesion. However, the exact etiology of psoriasis is unknown. Abnormalities in essential fatty acid metabolism, free radical generation, lipid peroxidation, and release of lymphokines have been proposed. *Objective*. Our purpose was to evaluate the plasma lipids and oxidized low-density lipoprotein accumulation in psoriatic skin lesion in order to ascertain the possible participation of oxidative stress and oxidative modification of lipids in pathogenesis of psoriasis. *Methods*. The study group included 84 patients with psoriasis, and 40 sex- and age-matched healthy volunteers. Blood lipid profile was determined. Psoriatic and nonlesional skin samples of psoriatic patients were evaluated for the presence of oxidized low-density lipoprotein by using an immune-fluorescent staining method. *Results*. The mean levels of lipids (total cholesterol, triglyceride, and LDL cholesterol) in patients with psoriasis were found to be significantly higher than those of healthy subjects. Psoriatic skins were shown positive oxidized low-density lipoprotein staining. There was no staining in nonlesional skin samples of the same individuals. *Conclusion*. Lipid peroxidation mediated by free radicals is believed to be one of the important causes of cell membrane destruction and cell damage. This study shows for the first time the accumulation of oxidized low-density lipoprotein in psoriatic skin lesion. We believe that accumulation of ox-LDL in psoriatic skin may have an important role in the immune-inflammatory events that result in progressive skin damage.

## 1. INTRODUCTION

The etiology of psoriasis is unknown, but genetic, metabolic, and immunologic mechanisms have been proposed [1]. It is known that psoriasis can occur due to abnormalities in essential fatty acid metabolism, lymphokine release, free radical generation, and lipid peroxidation. Alterations in plasma lipid and lipoprotein composition including a tendency toward an increase in total cholesterol (TC) and triglyceride (TG) and decrease in high-density lipoprotein cholesterol (HDL-C) levels suggest that psoriasis may associate with the disorders of lipid metabolism [2, 3]. Healthy skin secretes 85 mg of cholesterol within 24 hours whereas a psoriatic patient loses 1–2 grams of cholesterol with scales during that time [1].

The morphology of psoriatic skin is characterized by epidermal thickness and parakeratosis, a pronounced dermal vascular plexus, and the presence inflammatory cells in the superficial dermis and epidermis. Increased polymorph nuclear leukocyte levels damage surrounding tissue by releasing reactive oxygen species produced via NADPH oxidase/myeloperoxidase and proteolytic enzymes. Increased production of oxygen metabolites is a common feature of most human diseases including psoriasis and it usually triggers an upregulation of the antioxidant capacity, which is overwhelmed. When the oxidative stress develops, it leads to the oxidative damage of lipids and proteins [2, 4, 5]. Oxidation of the low-density lipoproteins (LDL) results in the production of modified LDL. One of the major and early lipid peroxidation products is oxidized low-density lipoprotein (ox-LDL) [6].

High titers of autoantibodies against ox-LDL have been reported in patients with psoriasis. The level of autoantibodies against ox-LDL has been suggested to reflect the in vivo oxidation of LDL [7, 8]. The presence of ox-LDL accumulation in psoriatic skin sample has not been shown before. The current study has been designed to evaluate the presence of ox-LDL accumulation in skin biopsy materials of psoriatic patients. We have also measured plasma lipids.

## 2. MATERIALS AND METHODS

### 2.1. Study groups

This prospective study was performed in the psoriatic patients attending the dermatology outpatient clinic of Zonguldak Karaelmas University Hospital. Eighty four psoriatic patients who were diagnosed clinically and histopathologically by the Department of Dermatology and a total of 40 age-and sex-matched healthy controls recruited from the general population as a control group were enrolled in the study. All patients had apparent psoriatic lesions, but no erythroderma or generalized pustulosis. They had not taken any medical treatment before. The patients with secondary hyperlipidemia such as chronic renal insufficiency, nephrotic syndrome, hypothyroidism, diabetes mellitus, obstructive liver disease, and the connective tissue disease were excluded. All patients and controls were included in the study after giving an informed consent. The Ethics Committee of Zonguldak Karaelmas University approved the study.

The height and weight of all subjects were recorded and their body mass indexes were calculated as weight (kg)/height (m^2^).

### 2.2. Determination of lipids and lipoproteins

Blood samples were taken after a 12-hour overnight fast and sera were separated by low-speed centrifugation for 15 min. The levels of serum TC, HDL-C, LDL cholesterol (LDL-C), and TG were determined by enzymatic methods using a Roche Cobas Integra 800 autoanalyzer.

### 2.3. Ox-LDL immune-fluorescent staining method of the skin biopsy materials

The skin punch biopsy specimens were collected from both lesional and nonlesional skin of 84 psoriatic patients. Uninvolved abdominal area was used as nonlesional skin sample. The presence of ox-LDL in biopsy materials of psoriatic individuals was evaluated using an immune-fluorescent staining method. The slides were prepared from biopsy sections, which were cut at 7-micron thickness. Slides were further divided into two pieces; one was used for the test and the other was used for negative control. Thirty *μ*l anti-oxidized LDL IgG solution (mouse IgG2 antibodies, AntibodyShop, Copenhagen, Denmark) as primary antibody was added only on test slides, and the control slides were manipulated only with the same amount of phosphate buffered saline solution (PBS). After 30 minutes of incubation in a humid chamber at room temperature, both the control and test slides were washed with (PBS), and 30 *μ*l FITC (fluorescent isothiocyanate)-labeled goat anti-mouse IgG (Chemicon International, California, USA) was administered as a conjugate substance. For a further 30 minutes, the slides were kept and incubated at room temperature, and then washed with the standard PBS solution. After open-air drying, slides were examined under fluorescent microscopy at 100X magnification (LEICA DMRX, Wetzlar, Germany).

### 2.4. Statistical analysis

Data were expressed as the means and standard deviations (SD). Differences in continuous variables between the patient and the control groups were analyzed using the Student *t*-test. Mann-Whitney *U* test was used to compare nonparametric variables between two groups. SPSS Windows release 11.5 was used. All values were expressed as mean ± standard deviation (SD) unless otherwise stated. Statistical significance level was set to .05 for all calculations.

## 3. RESULTS

Table 1 illustrates the clinical and demographic characteristics of the study population. There was no statistically significant difference between psoriatic patients and the controls considering age, sex, weight, height, or BMI (*P* > .05).

The levels of the lipid parameters are shown in Table 2. TC, TG, and LDL-C levels were significantly higher, but HDL-C levels were lower in the psoriatic patients than in the control subjects. However, gender-related results were completely different. HDL-C levels were significantly lower in the female psoriatic patients than in the female control subjects (*P* < .05). There was no statistically difference for TC, TG, and LDL-C levels between the psoriatic and the control subjects in the female group (Table 3). TC, TG, and LDL-C levels were significantly higher in the male psoriatic patients than in the male control subjects (*P* < .05). There was no statistically difference in HDL-C levels between the psoriatic and the control subjects in male group (Table 4).

**Figure 1:**
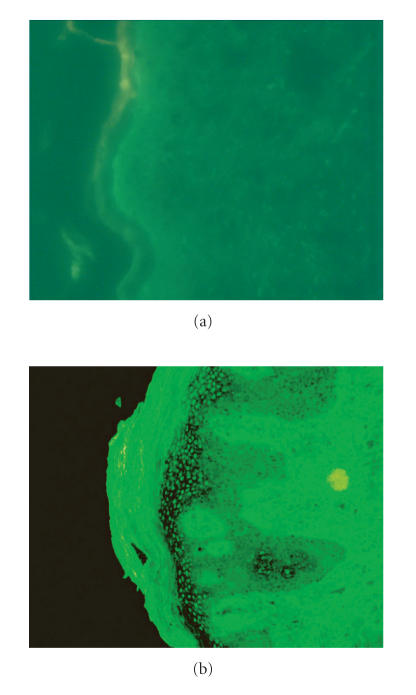
(a) Fluorescent microscopic view of the nonlesional skin biopsy material of psoriatic patient. There is no fluorescent staining. (b) Fluorescent microscopic view of the psoriatic skin biopsy material of the same patient. There is positive fluorescent staining which is the accumulation areas of oxidized low-density lipoprotein (X100).

We did not observe any positive immune-fluorescent staining in the nonlesional skin biopsy materials of the psoriatic patients. Significant positive immune-fluorescent staining was observed in the psoriatic skin biopsy materials. The dense cellular staining was observed in upper epidermis (Figure 1).

## 4. DISCUSSION

Among the many studies on serum lipid values in psoriasis, conflicting results have been reported. In studies on serum TC levels in psoriatic patients, high [2, 9, 10], low [1, 11], and normal [12, 13] values have all been reported. In our study, we found significantly higher levels of TC values in the psoriatic patients (*P* < .05). As for serum LDL-C levels, high [2] or normal [1, 10, 12] values have also been reported in psoriasis. We found that LDL-C values in the patients with psoriasis were significantly higher than the control group (*P* < .05). Normal [9, 10, 13] and low [1, 2] serum levels of HDL-C have been detected. In our study HDL-C levels in psoriatic patients were significantly lower than the control group (*P* < .01).

The same controversy exists regarding serum TG levels, high [1, 2], low [11], and normal [9, 10, 12] values have also been reported in psoriasis. We found that TG values in the psoriatic patients were significantly higher than the control group (*P* < .001).

The variety of data presented may be due to the fact that the patients included in statistical analyses suffer from different forms of psoriasis such as erythroderma and they undergo various treatments. Our patients had no erythroderma or generalized pustulosis. Furthermore they had not taken any medical treatment.

Our results were strongly related to the gender of the patients. TC, TG, and LDL-C levels were significantly higher in the male psoriatic patients than in the male control subjects. There was no statistically difference in the female groups. Only for the females, HDL-C levels were lower in the psoriatic patients than in the control subjects. There was no difference in the male groups for HDL-C levels. Considering the results of our research we would suggest analyzing the lipid profiles separately in males and females.

The relationship between augmented LDL-C level and psoriasis is unclear. However, ox-LDL may be more illuminative than native LDL. Native LDL is only taken up modestly by macrophages, whereas modified LDL is rapidly taken up via scavenger receptors [14]. The major modification of LDL particles in vivo is believed to be the oxidation of both its lipid and protein components [15]. Oxidized or modified LDLs are the main subjects of many inflammatory conditions such as atherosclerosis. The oxidized LDL hypothesis is discovered by Goldstein et al. [16] and modified by Steinberg et al. [17]. The current oxidative modification or stress hypothesis of atherosclerosis predicts that LDL oxidation is an early, essential event in atherosclerosis and that ox-LDL does contribute to both initiation and progression of atherosclerosis [18]. The oxidative modification hypothesis focuses on the concept that LDL in its native form is not atherogenic. The presence of ox-LDL in atherosclerotic lesions has been studied using antibodies that recognize specific epitopes on ox-LDL, which are not present in its native, nonoxidized, form. These antibodies avidly stain atherosclerotic lesions in humans with no demonstrable staining in normal arteries [19].

A typical feature of atherosclerosis is the accumulation of oxidatively modified LDLs within plaques. Also these lipoproteins are considered to contribute to the inflammatory state of atherosclerosis and to play a key role in its pathogenesis [20]. The cellular uptake of ox-LDL leads to the generation of reactive oxygen species (ROS) [21]. ROS are potentially very harmful substances, because they can react with proteins, DNA, or lipids. In other words, the accumulation of ox-LDL can be starter of oxidative stress.

The importance of this manuscript is to show the existence of ox-LDL in psoriatic skin. This study shows for the first time the accumulation of oxidized low-density lipoprotein in psoriatic skin lesions by direct immune-fluorescent method. Ox-LDL or anti-ox-LDL antibody levels can be measured in blood samples or body fluids by conventional biochemical and immunological methods. Especially, the level of anti-ox-LDL has been suggested to reflect the in vivo oxidation of LDL. The studies of Vanizor Kural [3] and Örem [7] have demonstrate the existence of these antibodies in psoriasis. The level of anti-ox-LDL antibody was positively correlated with TC and negatively correlated with HDL-C in their studies. In our study, we detected accumulation of ox-LDL in psoriatic skin. This accumulation is markedly increased especially in upper epidermis. The upper-epidermal cells had dens cellular staining with anti-ox-LDL antibodies. Basal layer of epidermis was not stained. We did not observe any positive immune-fluorescent staining in the nonlesional skin biopsy materials of the psoriatic patients. Leren et al. have observed that tissue-cultered skin fibroblasts from psoriatic patients have reduced LDL receptor activity [22]. Their study has no difference in LDL receptor activity between involved and uninvolved skin from our psoriasis patients. In our study, we only focused on the epidermal layer of involved and uninvolved skin from psoriatic patients. We detected accumulation of ox-LDL in the upper epidermis of the involved skin from the psoriatic patients. Ox-LDL can use native LDL receptor. However, it has a high-affinity advantage than its native form.

In conclusion, ox-LDL is an important marker of oxidative stress and lipid peroxidation process. Importantly, ox-LDL, by itself, may induce inflammation [23]. This capability may directly affect psoriatic epidermis and we believe that the accumulation of ox-LDL in the psoriatic skin may have an important role in pathogenesis of psoriasis.

## Figures and Tables

**Table 1:** The clinical and demographic characteristics of the psoriatic patients and the control group.

	Patients	Controls	*P*
	(*n* = 84)	(*n* = 40)	

Age (y) (median) (range)	39 (17–58)	36 (19–55)	> .05
Gender (M/F)	41/43	20/20	> .05
Height (cm)	172 ± 01	167.5 ± 9.5	> .05
Weight (kg)	75 ± 03	70.7 ± 12.0	> .05
BMI (kg/m^2^)	25.2 ± 5.1	25.3 ± 4.8	> .05

BMI: body mass index.

**Table 2:** Plasma lipids and lipoproteins in patients with psoriasis and control subjects.

	Patient	Control	*P*
	(*n* = 84)	(*n* = 40)	

TC (mg/dl)	183, 51 ± 13, 19	169, 45 ± 24, 02	.030*
TG (mg/dl)	124, 17 ± 58, 57	85, 15 ± 40, 98	.001*
LDL-C (mg/dl)	108, 58 ± 32, 76	96, 30 ± 25, 65	.031*
HDL-C (mg/dl)	48, 57 ± 12, 88	56, 18 ± 15, 30	.005*

*Statistically significant.

**Table 3:** Plasma lipids and lipoproteins in patients with psoriasis and control subjects for females.

	Patient	Control	*P*
	(*n* = 43)	(*n* = 20)	

TC (mg/dl)	179, 27 ± 43, 72	166, 55 ± 23, 43	.268
TG (mg/dl)	102, 59 ± 37, 88	85, 00 ± 41, 49	.075
LDL-C (mg/dl)	102, 65 ± 35, 20	94, 60 ± 21, 70	.457
HDL-C (mg/dl)	52, 18 ± 13, 81	61, 45 ± 15, 22	.021*

*Statistically significant.

**Table 4:** Plasma lipids and lipoproteins in patients with psoriasis and control subjects for males.

	Patient	Control	*P*
	(*n* = 41)	(*n* = 20)	

TC (mg/dl)	188, 08 ± 28, 43	172, 35 ± 24, 85	.039*
TG (mg/dl)	146, 30 ± 67, 64	85, 30 ± 41, 55	.001*
LDL-C (mg/dl)	111, 34 ± 29, 59	98, 00 ± 29, 56	.031*
-C (mg/dl)	45, 06 ± 11, 00	50, 90 ± 13, 82	.077

*Statistically significant.
